# Dynamic protrusive cell behaviour generates force and drives early matrix contraction by fibroblasts

**DOI:** 10.1016/j.yexcr.2007.07.040

**Published:** 2007-12-10

**Authors:** Annegret H. Dahlmann-Noor, Belen Martin-Martin, Mark Eastwood, Peng T. Khaw, Maryse Bailly

**Affiliations:** a Division of Cell Biology, UCL Institute of Ophthalmology, 11-43 Bath Street, London EC1V 9EL, UK; b Ocular Repair and Regeneration Biology, UCL Institute of Ophthalmology, 11-43 Bath Street, London EC1V 9EL, UK; c School of Biosciences, University of Westminster, 115 New Cavendish Street, London W1W 6UW, UK

**Keywords:** Matrix contraction, Collagen gels, Force, Protrusive activity, Live 3D microscopy

## Abstract

We investigated the cellular mechanisms underlying force generation and matrix contraction, using human corneal, Tenon's and scleral fibroblasts in a standard collagen matrix. We used timelapse light and confocal reflection microscopy to analyse concomitantly cell behaviour and matrix remodeling during contraction and devised a novel index to quantify dynamic cell behaviour in 3D. Based on the previously described culture force monitor, a novel simultaneous imaging and micro-culture force monitor system (SIM–CFM) was developed to measure the mechanical strain generated during matrix contraction whilst simultaneously recording cell and matrix behaviour. Ocular fibroblasts show marked differences in macroscopic matrix contraction profiles, with corneal fibroblasts inducing the strongest, and scleral the weakest, contraction. We identified four factors that determine the early matrix contraction profile: 1) cell size, 2) intrinsic cellular force, 3) dynamic cell protrusive activity and 4) net pericellular matrix displacement. Intrinsic cellular force and dynamic activity appear to be independent unique characteristics of each cell type and might serve as predictors of matrix contraction. The identification of these factors raises the fundamental new possibilities of predicting the ability of tissues to contract and scar and of modulating tissue contraction by targeting intracellular pathways linked to protrusive activity and force generation.

## Introduction

Tissue contraction plays an important role in diverse disease processes, such as scarring after injuries or surgery, as well as in idiopathic fibrosing conditions. In the eye and its surrounding tissues, the processes of wound healing and scarring are particularly relevant as they cause severe and blinding eye conditions [1]. A commonly used *in vitro* model of tissue contraction is the fibroblast-populated collagen matrix [2]: liquid collagen is added to a suspension of trypsinised fibroblasts; when the pH of the solution is neutralised, the collagen polymerises, with the fibroblasts dispersed throughout the resulting gel-like matrix. The cells contract the matrix down to a fraction of its original size, with the speed of contraction depending on cell type, density and collagen concentration. Both *in vitro* and *in vivo*, the cellular mechanisms of matrix contraction are still not fully resolved. Two main mechanisms have been proposed to account for tissue contraction: traction induced by cell migration or locomotion, and increased cell contractility through differentiation into α-smooth muscle actin expressing myofibroblasts. The involvement of cell locomotion is supported by evidence that, during cell spreading and migration, fibroblasts generate sufficient force to bend individual collagen fibres bound to their surface [3–6]. Initially only collagen fibres in the immediate vicinity of the cells are moved, but as the fibres are linked, tractional forces are propagated throughout the matrix, resulting in global remodelling and contraction [7–10]. However, it is unclear whether these tractional forces might be sufficient to induce *in vivo* wound closure as well as *in vitro* matrix contraction [11], and further experiments have suggested that net cell locomotion actually results in the release of tension within the matrix rather than local contraction [4,11,12].

Another possible mechanism accounting for matrix contraction, which centres around the development of a dedicated contractile phenotype, was derived from Gabbiani's original description, in 1971, of modified fibroblasts with smooth muscle like features in an experimental animal model of wound healing [13,14]. Subsequently, these modified fibroblasts were also found in pathological connective tissue conditions, such as fibrosis of parenchymal organs, fibromatosis and stromal reaction to tumours [15]. Today, the presence of α-smooth muscle actin (ASMA) in stress fibres is considered to be the main defining characteristic of this cell, named “myofibroblast” by Gabbiani's group [13,15,16]. However, *in vitro* the transformation from fibroblast to myofibroblast only occurs under special conditions and requires the presence of transforming growth factor beta (TGF-β), tension and time [17–20]. The usual delay between TGF-β stimulation and demonstration of ASMA incorporation into stress fibres is 72 h, although mRNA expression presumably occurs much earlier [21,22]. Myofibroblast transformation is therefore unlikely to explain early matrix contraction or contraction in the absence of tension, such as the contraction of free floating matrices *in vitro* and early *in vivo* wound closure [11,23].

Recently, a third mechanism of cell-induced traction on matrix has emerged: traction by cell protrusions not associated with net cell locomotion. Following earlier work describing how fibroblasts exert tractional forces onto the surrounding matrix [3], studies have shown that non-motile cells, through the dynamic extension and retraction of pseudopodial extensions, could generate local tension in the matrix leading to contraction [4,9,10,12]. Fibroblasts can also displace individual collagen fibres placed on their upper surface using protrusions and retractions in a typical “hand-over-hand” mechanism [6]. Chemical agents that lead to actin filament disassembly or block myosin activity inhibit matrix re-arrangement, indicating that the players involved in migration are also at the basis of traction without migration [10]. A link between force development and spreading morphology has also been observed in fibroblasts contracting a collagen–glycosaminoglycan matrix, where matrix deformation and force development are associated with cell elongation after trypsinisation, but not migration [24]. However, most of these studies involve the use of cells plated on top of collagen matrices rather than within [4,6,12,25] and to date, there is no direct evidence that links this dynamic cellular behaviour of protrusive activity to force generation and matrix contraction in a three-dimensional model. Furthermore, there is as yet no way of predicting a cell's ability to contract a 3D matrix if they do not express the typical myofibroblastic phenotype, although clearly *in vivo* some tissues are more prone to contraction and scarring than others. We used the classical *in vitro* model of fibroblast-mediated 3D collagen matrix contraction to identify the links between cell morphology and behaviour and the contraction developed, using fibroblasts isolated from different parts of a single organ, the eye, which present distinct propensities to contract and scar. Earlier studies using force measuring devices for cells in 3D matrix correlated cell morphology with force measurements at defined, static time points [24,26]. Based on one such tool [27], we have developed a novel device, the SIM–CFM, which allows simultaneous microscopical cell and matrix visualisation and force measurements. Using this setup, we demonstrate that the basic mechanism of force generation by fibroblasts embedded in three-dimensional collagen matrix is linked to “on the spot” protrusive and retractile activity and that the ability of cells to contract collagen matrices is defined by their intrinsic dynamic activity level and contractile force.

## Materials and methods

### Cell culture

Primary fibroblast cultures were established from cornea, episclera (Tenon's layer) and sclera of human donor eyes by dispase digestion as previously described [28], according to the tenets of the Declaration of Helsinki and local ethics approval. Corneal and Tenon's fibroblasts from three different donors and scleral fibroblasts from one donor were used. Cells were maintained in complete medium (DMEM with 10% Foetal Bovine Serum [FBS] and 100 U/ml penicillin, 100 mg/ml streptomycin and 2 mM l-glutamine, Invitrogen) in tissue culture incubators with 5% CO_2_ and 95% humidity. Experiments used cells of passage 2 to 10. For live imaging experiments, DMEM was replaced by Leibovitz L-15 medium 1× with l-glutamine, without phenol-red (Invitrogen). For experiments in serum-free medium (control starved cells), the FBS was replaced with 0.7% BSA (Bovine Serum Albumin, Fisher Scientific) to maintain the osmotic pressure.

### Collagen contraction assay

Collagen matrices were prepared following a modification of a previously described protocol [29]. In brief, cells were trypsinised, counted, and where appropriate, cell viability counts were performed using trypan blue (Invitrogen). Cell suspensions containing the desired number of cells were diluted in PBS and centrifuged. The cell pellet was resuspended in pure FBS or serum-free medium with 0.7% BSA. Concentrated medium (350 μl l-glutamine, 900 μl sodium bicarbonate solution 7.5% [Invitrogen], 3.5 ml Dulbecco's modified Eagle's medium 10× [Sigma]) and liquid rat tail collagen type I in acetic acid (First Link, UK) were added for a final collagen concentration in the matrix of 1.5 mg/ml and the pH was rapidly adjusted to 7 using NaOH 1M. Two standard cell concentrations were used: a low cell density of 40,000 or 60,000 cells/ml matrix volume to study global matrix contraction and dynamic behaviour of individual cells, and a high cell density of 10^6^ cells/ml matrix volume for force measurement experiments. Matrices were either cast in the shape of 150 μl buttons in the 14 mm diameter central wells of 35 mm MatTek tissue culture dishes (MatTek Corporation, Ashland, USA), or as 2 ml blocks in 14 × 22 mm custom-made rectangular moulds bearing 2 Vyon™ floatation bars (Porvair, UK) and metal hooks at their extremities (for culture force monitor experiments). After 30 min of polymerisation at 37 °C in a tissue culture incubator, the matrices were manually detached from the wells or moulds and floated in medium. Whole matrix contraction was monitored by digital images taken immediately following release of the polymerised matrices and then daily for 7 days. Images were imported into Image J software, and the matrix area was measured with reference to the outline of the well. Matrix surface area was normalised to the area calculated at T0 and expressed as percentage of the initial surface area, according to the following formula: *A* (*t*_*n*_) in % = 100 − (100 × *R*_*tn*_^2^ / *R*_*t*0_^2^) with *A* = area, *R* = gel radius, *t*_*n*_ = time point between 24 and 168 h after matrix preparation. Each experiment was performed as triplicates of matrices under different conditions.

### Quantitation of total actin and ASMA

Fibroblasts were seeded in collagen gels as above and the gels were incubated in medium plus 10% serum. The gels were digested with 0.05% Collagenase D in PBS containing Ca^+ 2^ and Mg^+ 2^ with gentle shaking for 20 min at 37 °C immediately after collagen polymerisation (time 0; approximately 30 min following seeding) and after 24 h. The cells were counted and lysed with Tris 50 mM containing 0.2% of SDS (100 μl of solution/10^6^ cells) for 30 min on ice. Cell extracts were clarified by centrifugation at 14,000 rpm for 5 min at 4 °C. Protein amount was estimated using the DC Protein Assay from Biorad according to the manufacturer's instructions. Cell lysates were prepared in Laemmli buffer and boiled for 5 min. Rabbit muscle actin (10 mg/ml, Cytoskeleton) was loaded as a standard to determine total actin concentration per cell. Proteins were separated by SDS 10% PAGE and transferred to PVDF membrane. After blocking with 1% of BSA in TBS-T buffer (20 mM Tris–HCl, pH 8.0, 150 mM NaCl, 0.1% Tween 20) for 1 h at room temperature, blots were incubated overnight with mouse monoclonal anti-actin antibody (Chemicon International) or mouse monoclonal anti-α smooth muscle actin (Sigma). Blots were washed three times for 10–15 min each with TBS-T followed by 1 h incubation with anti-mouse–HRP conjugate (Dako). After final washes, the blots were revealed using an ECL detection system (Amersham GE Healthcare). Semi-quantitative analysis of net total actin and relative A-SMA protein concentration per cell was performed using the gel analyser module in Image J following scanning of the developed films.

### Microscopy

Live cell differential interference (DIC) imaging within the matrices was performed on a Zeiss Axiovert 100M using standard objectives (Plan Neo-Fluar 10× 0.3NA, 20× 0.5NA and Fluar 40× 1.3NA, Zeiss) and a × 60 long working distance air objective (Olympus LCP Plan FI 60×, NA 0.70, infinity corrected, with correction collar, Olympus Optical, UK). Images were taken using a CCD camera coupled to an OpenLab-driven image acquisition system (Improvision). During live timelapse microscopy, the medium in the culture dishes containing the specimens was covered with a layer of heavy mineral oil (Sigma) to prevent evaporation. Collagen matrix fibrils were imaged by confocal reflection microscopy as previously described [30], using the 60× objective above on a Zeiss Axiovert S100 TV/Biorad Radiance 2000 confocal microscope, and DIC images of cells were acquired in parallel. Images of full cell volumes were acquired every 10 or 15 min for 15 h in *z*-stacks with 3 μm steps in a fixed *x*–*y* plane for quantitation of fibre movement and cell protrusive and retractile activity.

### Quantitation of cell volume, protrusive activity and matrix displacement

To evaluate cell volume, digital pictures were taken of cells in suspension. The cell diameter of 20 to 40 cells of each type was measured on two axes on these digital photos using Image J (http://rsb.info.nih.gov/ij/), and the cell volume was calculated from the cell diameter, using the formula for the calculation of the volume of a sphere: *V* = 4/3*πr*^3^ (where *r* = radius). The maximum cell length (longest line from tip of one protrusion to tip to another protrusion) was measured on compressed *z*-stack images in Image J.

**Fig. 1:**
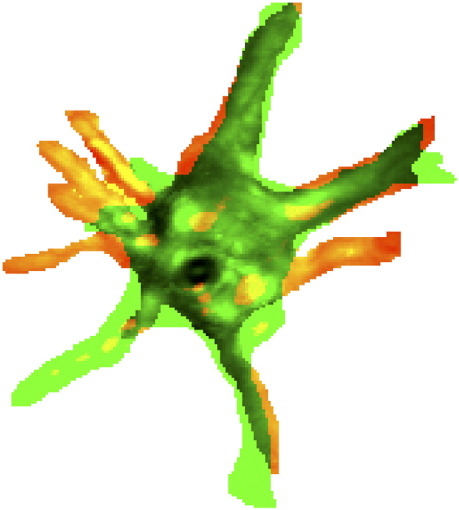
The dynamic index (DI) as a measurement of the cell's dynamic behaviour in 3D. The DI measures the proportion of cell area that has been remodelled between two time points. Shown is a merge of binary images corresponding to the cell area at time *t* (red) and *t*_+ 15_ min (green). Purely green areas represent newly formed protrusions, whilst purely red areas reflect processes that have been retracted between the first and the second time point. The corresponding DI per time frame is defined as: (areas of new protrusions + areas of retractions) / cell area (see Materials and methods for details).

To quantitate cell dynamic activity, we devised a novel parameter, the dynamic index (DI), which quantifies the total area of dynamic activity (i.e. protrusion plus retraction) as a function of the cell area. Confocal *z*-stacks acquired every 15 or 20 min were imported into Image J software and compressed into single images using the maximum intensity projection setting. The outline of individual cells was manually traced to allow accurate image thresholding. Cell area and changes in cell shape (protrusion-green and retraction-red, Fig. 1) were quantified using a modification of the colocalisation module of Metamorph, and cell dynamic activity over time or “dynamic index” (DI) was calculated for each time frame as: (areas of protrusions + areas of retractions) from *t* to *t*_+ 15_ or *t*_+ 20_ divided by the cell area at *t*. As values for area change did not significantly differ between timelapse series acquired every 15 or 20 min, data were pooled. Collagen fibre displacement was quantified by manually tracking individual fibres on subsequent time points using Image J software. The first hour of imaging was excluded, as small vertical drifts of the specimen during initial collagen matrix hydration prevented accurate maintenance of the same focal plane.

### Force measurement and application of external tension

**Fig. 2:**
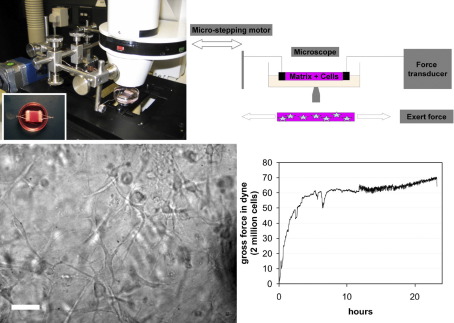
The SIM–CFM allows simultaneous imaging of cells and matrix and measurement of force generation. Top left: CFM setup on Zeiss Axiovert epifluorescence microscope. Inset: freshly cast collagen matrix with embedded floatation bars and hooks to connect it to CFM suspension wires. Top right: working principles of the SIM–CFM. As fibroblasts contract matrix, a flexible metal strip connected to one of the embedded floatation bars is deflected. A force transducer converts the mechanical input into an electrical signal, which is recorded as force measurement on a PC. Cell behaviour and matrix interaction are visualised using light or confocal microscopy. Bottom: concomitant live cell imaging and contraction measurement on the SIM–CFM. HCF were seeded in collagen gels at a density of 10^6^ cells/ml and placed on the SIM–CFM setup. Left image shows the cells in the gel 16 h after matrix preparation, as imaged live during contraction using DIC on an epifluorescence microscope with × 20 objective. Scale bar 50 μm. Right: corresponding force recording acquired simultaneously.

Based on the previously described tensioning culture force monitor (t-CFM) [27,31], we developed a novel device allowing simultaneous microscopic imaging, measurement of force generation by cells in collagen matrix and application of external tension: the SIM–CFM (simultaneous imaging and culture force monitor, Fig. 2). A force transducer was mounted onto a Zeiss Axiovert 100 microscope stage, and a microstepping motor (Micromech systems ltd, Braintree, Essex, UK, software-operated using XWare 6.0.4, Parker) was used to apply tension onto specimens placed on the microscope stage. Both force transducer and motor were connected to a personal computer and operated via software control (PicoLog, Pico Technology Ltd, and XWare 6.0.4, Parker). The system was complemented by a custom-built microscope incubation chamber and a humidifier (Zeiss). As the fibroblasts contract the matrix, the transducer converts the mechanical deflection signal into an electrical one, which is recorded on a PC, using the software package PicoLog™. Force measurements without microscopy were obtained by placing the culture force monitor (on the microscope stage) in a tissue culture incubator (37 °C, 95% humidity, 5% CO_2_) and recording force development over 16 h after matrix preparation. For simultaneous imaging, the SIM–CFM stage was mounted in an incubation chamber (37 °C, 70% humidity, no CO_2_) on a confocal or epifluorescence microscope as above. Calibration was performed as previously described [27]. Net contractile force was calculated by subtracting the force measured during baseline experiments of contraction of matrices devoid of cells from the gross force recorded during contraction of 2 ml matrices seeded with 10^6^ cells/ml and dividing the resulting value by 2 to obtain the net force per million cells.

## Results

### Ocular fibroblasts differ in matrix contraction efficiency

**Fig. 3:**
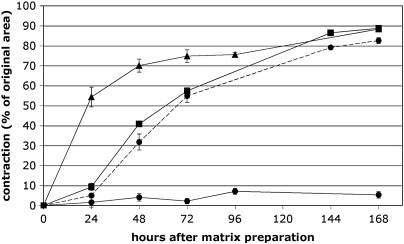
Ocular fibroblasts vary in their ability to contract collagen matrices. Collagen matrices were seeded with HCF (triangles) or HTF (squares) at a density of 40,000 cells/ml, and with HSF (circles) at densities of 40,000 (continuous line) and 3 million cells/ml (dotted line). The matrices were released from the casting well 30 min after preparation and were freely floated in DMEM with 10% FBS. Matrix contraction is expressed as percentage of the original gel area. Data show mean ± SEM, from 2 sets of triplicate matrices for HCF and HTF, and 1 set of triplicate matrices for HSF.

Ocular fibroblasts are involved in numerous conditions that can affect vision, but details of how these cells mediate wound healing and scarring events are not known. In particular, potential differences between ocular fibroblasts from different parts of the eye have not been studied systematically. We therefore began our investigation by studying the remodelling and contraction of collagen matrix by three types of ocular fiboblasts, human corneal, Tenon's and scleral fibroblasts (HCF, HTF, HSF, Fig. 3). HCF contracted the matrix by about 55% within the first 24 h, and the contraction reached a plateau at the maximum value after 72 h. HTF remodelled matrix more slowly: matrix contraction was only 10% after 24 h, but gradually increased until reaching maximal contraction similar to HCF after 6 days. The scleral fibroblasts used in this study were isolated from the anterior sclera, close to the corneal limbus. These cells were typically much smaller than the other two types of fibroblasts and maintained a rather rounded morphology within the collagen matrix as opposed to the spindle-shaped or stellar morphology of the HCF and HTF (Fig. 5). As expected from these observations, when HSF were seeded in the collagen matrices in numbers comparable to the HCF and HTF, they failed to significantly contract the matrix. However, matrix contraction profiles similar to those obtained with HTF were generated when the cell density in the matrix was increased by 75 fold (Fig. 3, dotted line).

### Dynamic “on the spot” protrusive activity is the main cellular behaviour in early matrix contraction

**Fig. 4:**
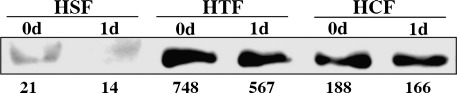
ASMA expression does not increase during early contraction. Collagen matrices were seeded with HSF, HCF or HTF and allowed to contract for 24 h in the presence of 10% serum. Cells were isolated from the matrix following collagen digestion immediately after gel polymerisation (30 min post seeding, 0d) or after 1 day (1d), and lysates were analysed by Western blot with anti-ASMA antibodies (HSF and HCF, 20 μg protein; HTF, 10 μg). The blots were analysed using Image J and the relative protein concentration per cell was calculated (indicated under the bands: intensity per cell, arbitrary units).

**Fig. 5:**
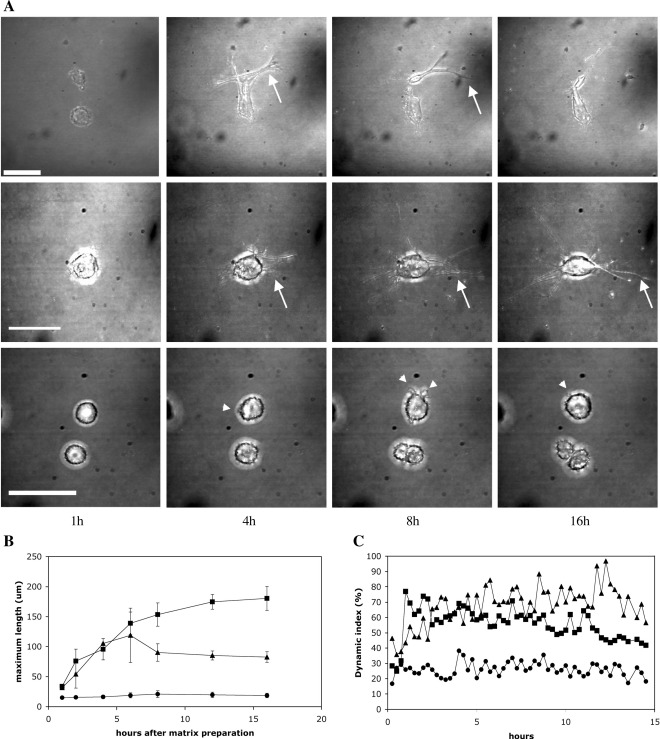
Ocular fibroblasts display different behaviour during establishment in the collagen matrix. Collagen matrices were seeded with 40,000 cells/ml. Matrices were released from the casting well 30 min after preparation, freely floated in L15 medium with 10% FBS and transferred to a confocal microscope. Starting 1 h after matrix preparation, timelapse *z*-stack phase contrast images of individual cells were obtained every 15–20 min for 15 h. *z*-Stacks were compressed into single projections for each time point. (A) Montage shows representative images of HCF (top), HTF (middle) and HSF (bottom) at selected time points. In the first hours after matrix preparation, HCF and HTF form progressively longer protrusions (arrows), whilst HSF remain mostly spherical with minute projections (arrowheads). Scale bars = 50 μm. (B) Variation in cell length during establishment in the collagen matrix. The maximum cell length (longest line from tip of one protrusion to tip to another protrusion) was measured in compressed *z*-stack images in Image J. The data shown are the means ± SEM for 3 HCF (triangles), 3 HTF (squares) and 2 HSF (circles). (C) Dynamic activity of the different types of ocular fibroblasts during establishment in the matrix. On the compressed projection images, the cell outline was tracked in Image J and changes in cell area analysed to calculate the dynamic index (Fig. 1 and Materials and methods). The dynamic index represents the fraction of cell area that is redistributed between time points. Data show the mean of 3 experiments for each cell type (HCF, triangles; HTF, squares; HSF circles; SEM range: 0.03–31%).

The first 24 h after matrix preparation saw the greatest differences between the contraction profiles of the 3 cell types. In order to rule out that these were simply due to differences in the actin content of the cells, we determined the net total actin concentration per cell, as well as their relative content in ASMA, using semi-quantitative Western blotting. HCF and HSF presented very similar actin concentrations (270 and 230 μM respectively), whilst HTF had significantly more actin at 460 μM per cell. All 3 cell types had very different relative contents in ASMA, with HSF being the lowest and HTF the highest, but there was no increase in the cellular content in ASMA after 24 h of culture in the gel (Fig. 4). Rather, there seemed to be a slight decrease compared to day 0 for all cell types. We thus set out to investigate in detail the behaviour of the cells within that phase and how it could relate to their contractile phenotype. In particular, we were interested in finding out whether cell migration across matrix might be the mechanism underlying matrix contraction [32]. We used confocal timelapse microscopy with simultaneous acquisition of phase contrast images to visualise the cells and reflection microscopy to visualise the matrix. Timelapse microscopy showed that, immediately after matrix preparation, cells were rounded and devoid of protrusions. In the following hours, cells extended and retracted protrusions of increasing length (Fig. 5A), reaching their fully spread morphology (as estimated by the maximum cell length) by about 6 h after seeding (Fig. 5B). In HCF and HTF, the overall cell shape changed from rounded to star or spindle shape. The HSF only extended short processes, and their cell shape remained mostly spherical. Regardless of the cell type, protrusion formation was found to be a highly dynamic process. Existing protrusions were continuously retracted, whilst new processes formed. Cell migration, on the other hand, was a rare event. Of 8 cells imaged for 16 h after matrix preparation (3 HCF, 3 HTF, 2 HSF), no cell migrated across the matrix. An additional 32 HCF and HTF were observed for 4 h each. Of these, only one cell showed net translocation within the matrix.

### Protrusive activity leads to matrix displacement

Measuring cell length at selected time points, although useful to judge the cell spreading, did not reflect the dynamic protrusive activity observed on timelapse movies, with continuing retraction of existing and formation of new protrusions. We therefore developed a new method to quantify cell protrusive activity, by digitally subtracting the area of a cell on a selected timelapse image from the area on the previous timelapse image. This results in two numerical values, one for the area of retracted protrusions and one for the area of newly formed protrusions. Based on these values and on total cell area on these images, we calculated a parameter reflecting overall protrusive activity, the “dynamic index” (DI), which we define as (areas of new protrusions + areas of retractions) / cell area for a given time frame. By quantifying the total area of dynamic activity (i.e. protrusion plus retraction) as a function of the cell area, the DI gives an accurate representation of how much of the cell area is being remodelled over a given period of time (Fig. 1). During the first 3 to 6 h after matrix preparation, all three cell types showed a rapid increase in dynamic activity as they spread through the matrix (Fig. 5C). Once the cells reached their optimal spread, the DI plateaued, albeit at different levels for the three cell types: HCF redistributed around 70–80% of their cellular area every 15 min, compared with 60% for HTF. The HSF showed a very low dynamic index, with barely 30% of the cell area being remodelled every 15 min. The dynamic index appeared to be a reproducible and intrinsic characteristic of each cell type, as exemplified by the consistency of multiple measurements by different experimentators in our laboratory (data not shown).

**Fig. 6:**
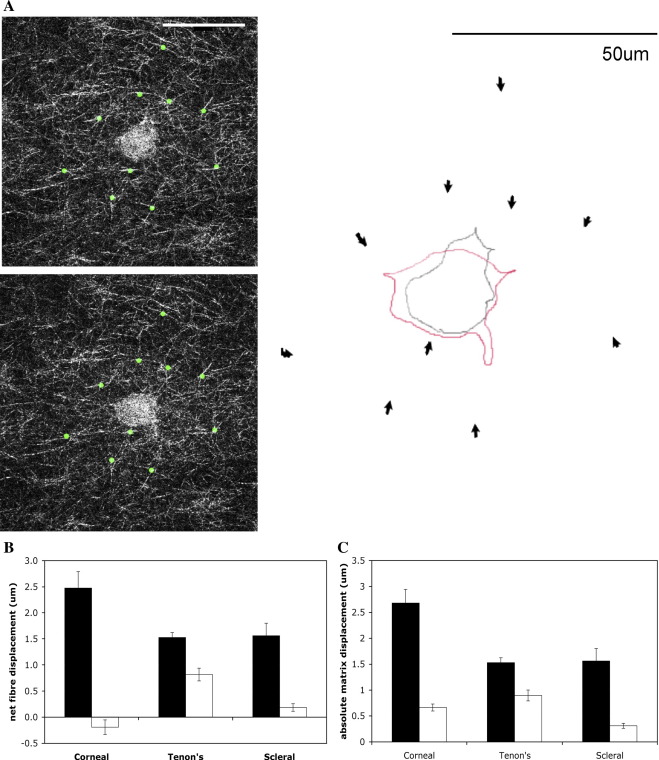
Protrusive activity is associated with traction on collagen fibres and net matrix displacement. (A) Left: Confocal reflection images showing cell and individual collagen fibrils acquired 1 (top) and 2 (bottom) h after matrix preparation with HTF. The displacement of 10 individual fibres (marked with green dots) was tracked in Image J. Right: Schematic representation of the movement of the fibres. Arrows indicate the direction of fibre displacement, with arrow length corresponding to the net displacement, in this example an average of 1.27 μm over 1 h. Superimposed the change in cell shape over the same time period (grey outline: first time point = 1 h after matrix casting, red outline: second time point = 1 h later). Note retraction of protrusion at the top and extension of new protrusion towards the bottom of the image. Scale bar: 50 μm. (B) Pericellular matrix displacement during early and late phase of fibroblasts establishment within the matrix. Net displacement of individual collagen fibres around the cells over the first hour following gel polymerisation and matrix release (black bars), and average per hour between 6 and 8 h post release (white bars). Fibre displacement towards the cell body or towards cell extensions was allocated a positive value, displacement away from cell extensions and cell body a negative value. Net displacement was calculated as the average of all measured values (± SEM) for 12 fibres tracked in timelapse series of three HCF and HTF and two HSF. (C) Variation in absolute amount of displacement of pericellular fibres. The values for fibre displacement measured in B were averaged as absolute values to evaluate the extent of fibre movement independently of the direction (towards the cell or away from the cell).

Whilst cell protrusive activity was the main cell behaviour observed during the first 24 h after matrix preparation, it did not in itself directly explain matrix contraction. In order to investigate whether there was a link between formation and retraction of protrusions and matrix contraction, we measured the displacement of individual collagen fibres in the immediate vicinity of cells at selected time points. The cell dynamic activity (protrusion and retraction) was associated with significant traction on collagen fibres, with individual pseudopods pulling fibres towards the cell body, resulting in net matrix displacement (Fig. 6A). To assess potential changes in matrix contraction intensity over time, we measured the fibre displacement between the 1st and 2nd (active cell spreading) and between the 6th and 8th hour (post-spreading plateau phase) after matrix preparation. In all three cell types, net matrix displacement was greater in the first 2 h after matrix preparation than in the plateau phase of dynamic activity (Fig. 6B). The lower net matrix displacement recorded at later time points was the result of a decreased ability to move the matrix rather than the cells displacing the matrix in opposite direction as the total matrix displacement was reduced for all 3 cell types (Fig. 6C).

### The early matrix contraction profile is linked to the level of intrinsic cellular contraction force

**Fig. 7:**
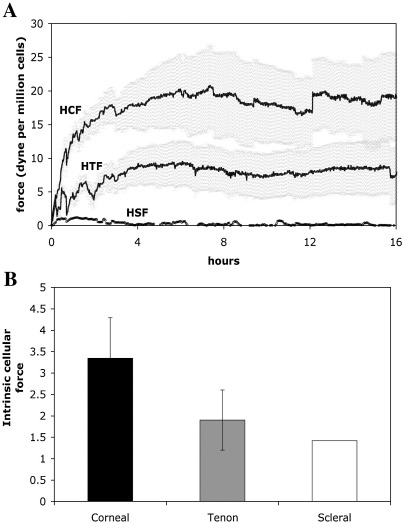
Force generation during early matrix contraction. (A) Collagen matrices were seeded with 10^6^ (HCF and HTF), or 7 × 10^6^ HSF fibroblasts per ml, floated in serum-supplemented medium and transferred to the culture force monitor stage. Cell-free matrices were prepared as negative controls to determine the baseline for the strain. The force associated with matrix contraction was recorded every second for 24 h, and measurements obtained over 1 min were averaged into one value. The average force reached after 7 h after matrix preparation (plateau phase, calculated from 6 to 13 h) in cell-free collagen matrices (5 experiments, total number of measurements recorded *n* = 1204) was subtracted from the values reached by the cell-seeded matrices (HCF: 6 experiments, *n* = 2166; HTF 7 experiments, *n* = 2527; HSF 1 experiment, *n* = 121), and the resulting figures corrected for the cell number to obtain the average maximum force generated by 10^6^ cells for each cell type. The graph shows the mean force and SEM over 16 h after matrix preparation. (B) Intrinsic cellular force. The force value measured at plateau phase for each cell type (7–13 h after matrix preparation) was divided by the mean cell volume (see Materials and methods) to obtain the amount of force per cell volume or intrinsic cellular force (nanodyne/μm^3^). Shown is mean ± SEM for HCF and HTF and single value for HSF.

In order to link protrusive activity and fibre displacement to global matrix contraction, we investigated the strain exerted by the cells onto their surrounding matrix during early contraction using a modified culture force monitor, the SIM–CFM (Fig. 2). The force development curves showed a rapid increase in force generation in the first 6 h after matrix preparation followed by a plateau (Fig. 7). The plateau values reached by the different types of fibroblasts differed markedly. The maximum force generated by the HTF (8.8 ± 3.2 dynes/10^6^ cells) is about a third of that of the HCF (20 ± 5.7 dynes/10^6^ cells), whilst the HSF hardly generated any force (around 2 dynes/10^6^ cells for the maximum, barely above background on average). During our experiments, we had observed that the different cell types differ greatly in cell size, with the HTF having about 76% (4600 ± 380 μm^3^) and the HSF about 23% (1360 ± 90 μm^3^) of the volume of HCF (6000 ± 530 μm^3^). As cell size might in part underlie the differences in force generation, we calculated the intrinsic cellular force per cell volume for each cell type (Fig. 7B). The values for intrinsic force lie within a similar range for all three types of fibroblasts (in the nanodyne/μm^3^ range), but with significant differences between each cell lines matching their macroscopic contraction profile.

## Discussion

The biological behaviour and molecular mechanisms underlying tissue contraction *in vivo* and matrix contraction in *in vitro* models are still controversial and, despite the recognised critical involvement of fibroblasts in the contraction process, we have as yet to identify parameters that could reliably predict one cell's contractile behaviour. We developed a novel device, the SIM–CFM, to analyse in detail and in real time the connections between the development of tensile strain in the matrix and the changes in cell morphology during contraction. Here we applied this system to the analysis of the behaviour of fibroblasts originating from three different tissues within the eye. Our work has uncovered two major novel findings: firstly, fibroblasts from different parts of the same organ not only differ in their matrix contraction profile, but can be characterised by an intrinsic level of actin dynamics and genuine contractile force, and secondly, early matrix contraction and force generation by fibroblasts are linked to the level of cell dynamic activity.

Previous studies have described the matrix contraction profiles of individual types of ocular fibroblasts, but direct comparison of published values is difficult because of the disparities of the experimental designs (different fibroblasts species, cell densities in the matrices, cytokines used to trigger contraction, types of matrices…etc.). Nevertheless, corneal fibroblasts in general appear to contract collagen matrices very rapidly [29,33]. This correlates well with clinical observations showing that corneal injury that reaches the stroma triggers a prompt activation of quiescent keratocytes into active fibroblasts [34]. Tenon's fibroblasts on the other hand displayed a slow start over the first 24 h followed by a sustained increase up to levels of contraction similar to those of corneals after 6 days, consistent with previous analyses [35]. Again, there might be a parallel to their behaviour *in vivo* as tissue contraction and scarring mediated by Tenon's fibroblasts, such as after glaucoma filtration, vitreoretinal or strabismus procedures, are commonly not acute events, but proceed long after surgery [36]. The scleral fibroblasts selected for this work were unusual in that their protrusions were rather short and thus served as negative control in terms of dynamic cell activity and contractile behaviour. Nevertheless, the poor ability of these cells to contract collagen matrix could easily be compensated for by increasing the cell density in the matrix, bringing them to a contraction profile identical to that of the Tenon's. This slow and sustained matrix remodelling response for the sclerals is consistent with previous work [37], as well as with the slow and gradual remodelling of the sclera observed clinically in problems such as axial myopia or elevated intraocular pressure in childhood glaucoma. Interestingly, the similarity in contraction profiles for the HTF and HSF is consistent with their common embryological origin, the mesoderm, whilst corneal fibroblasts more likely are derived from the neural crest [38,39].

The fact that different fibroblasts show marked differences in their contractile properties could have important repercussions for the modulation of wound healing, matrix remodelling and tissue scarring. Although the free floating collagen matrix model oversimplifies the dynamic cell–matrix interactions normally occurring in tissue, our results suggest that fibroblasts from different parts of the eye are intrinsically different in their behaviour. Indeed, the existence of significant differences in the contractile force displayed by the 3 cell types suggests an intrinsic difference in the cells' ability to generate force. This intrinsic contractile force appears to be predictive of the cell's ability to contract the matrix, but does not seem to be correlated to the total actin concentration within the cell or the amount of ASMA. Interestingly, the fact that even the poorly contractile scleral cells present a force and cellular actin concentration in the same range as those of the other types of fibroblasts is consistent with their ability to reach greater matrix contraction profiles when cell numbers are increased. Such variations between the levels of contraction forces for different types of fibroblasts with the same organ have not been reported previously, although one study compared Tenon's and skin fibroblasts and demonstrated a lesser contractile force in Tenon's fibroblasts, consistent with our results [32]. The fact that the intrinsic force is within the same range for all three cell types, despite their remarkably different morphologies and contractile behaviour, suggests that the intrinsic force is underlined by a common mechanism, most likely actin dynamics [10,40] and actomyosin contraction [41]. Indeed, actomyosin interaction is also the motor underlying extracellular matrix fibre displacement associated with “hand-over-hand” cycles of cell extension, matrix fibre attachment, retraction and fibre release [6], which leads to global matrix contraction through orthogonal amplification [9].

**Fig. 8:**
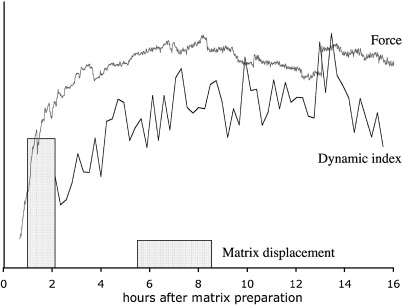
Profiles of dynamic cell protrusive activity, matrix displacement and force generation show similar profiles over time. Matrix displacement is higher at earlier time points and then decreases despite continued maximal force and protrusive activity, indicating that additional factors are at play. Averaged force curves (top line, from Fig. 6A), dynamic index values (lower line, from Fig. 4C) and matrix displacement (blocks representing absolute matrix displacement, from Fig. 5B) representative of corneal fibroblast superimposed into one diagram.

The second main finding of this work is that early matrix contraction and force generation are strongly linked to the level of cell dynamic protrusive activity, i.e. the continuous extension and retraction of dynamic cellular processes. The most marked differences in matrix contraction in our model occur within the first 24 h of matrix preparation, where “on the spot” protrusive activity is the main cell behaviour observed. We did not observe any significant cell migration during contraction, even at later time (this work and Martin-Martin et al., manuscript in preparation), nor did we notice the build up of a significant contractile-like phenotype with large ASMA-containing stress fibres (A. Dahlmann, personal observation and Ahir et al., manuscript submitted). Rather, our results support the concept of matrix contraction by non-migrating fibroblasts using the “locomotion” machinery for cell–matrix interaction and collagen fibre displacement [6,12,42]. Whilst it has been noted that initial matrix contraction is associated with cell “spreading” from a round to a branched phenotype [26,43], the study of the dynamic behaviour of cells in the context of matrix contraction has largely been confined to two-dimensional models, with fibroblasts seeded on top of a collagen layer [3,4,12,25]. Interestingly for both cells on top of the matrix [12] and within a 3D matrix [42], both the extension of cell protrusion and their partial retraction have been linked to collagen fibre displacement towards the cell and the generation of local tension. Although we have shown that the dynamic activity of the cells is associated with net displacement of collagen fibres towards the cell, at least during the spreading phase, we have not in this study examined in detail the displacement of the collagen fibres during the different phases of the dynamic protrusive activity. Nevertheless, our observations are consistent with the notion that the dynamic activity of the cells leads to local collagen remodelling through a combination of direct pulling through actomyosin contraction during retraction of the processes, and more subtle tension exerted on the matrix during the extension of the pseudopods through the formation of new adhesions in conjunction with periodic local actomyosin waves [4,42,44]. Interestingly, our quantitation of dynamic protrusive activity demonstrates that this constant cell shape remodelling through dynamic extension and retraction of protrusions persists, at a level characteristic of each cell (i.e. dynamic index), even after the cells have fully spread in the matrix. However, further remodelling of the matrix likely involves other mechanisms (Ahir et al., manuscript submitted), as we observed that the displacement of the pericellular collagen fibres is greatly reduced once the cells are fully spread and have reached the plateau value for their dynamic activity. As the dynamic index and the force reach an equilibrium value with similar kinetics, and this plateau phase corresponds to a decrease in local fibre displacement, our data suggest that the cells use a down-regulation of their adhesion to the matrix, rather than a decrease in their dynamic protrusive activity, to maintain homeostasis (Fig. 8; [25,31]). This type of regulation might also be involved in later stages of matrix contraction, as we have found that the dynamic index remains stable throughout the typical 7-day contraction assay (Martin-Martin et al., manuscript in preparation). Further work is needed to explore how temporal modulation of cell–matrix adhesions could fine-tune tensional homeostasis.

Overall, we have identified four factors, which influence the early matrix contraction profile of the three types of ocular fibroblasts we studied: 1. cell size, 2. intrinsic cellular force, 3. dynamic activity and 4. net matrix displacement. Of these, two main parameters seem to determine the matrix contraction profile characteristic for each cell type: intrinsic force and dynamic protrusive behaviour. Cells with high intrinsic force and high dynamic index (HCF) induce a rapid increase in matrix tension and contraction, and cells with low force and low dynamic index induce slower contraction profiles (HSF). Combination of lower force with high dynamic index (HTF) results in slower, but eventually equally effective matrix contraction, indicating that the intrinsic cellular force is the main predictor of speed of matrix contraction. By analysing in detail early events in the standard model of cell-mediated matrix contraction, we have been able to isolate specific parameters that underlie a cell's ability to contract collagen matrices and profile a general model of the early phases of contraction detailing the complex interactions between the cell dynamic activity, local matrix displacement, and force generation (Fig. 8). This work offers essential new insights into the mechanisms of cell-mediated matrix contraction and supports the novel idea that specific cellular parameters such as intrinsic contractile force and dynamic index can be used to predict a cell's ability to contract matrix. Exploring the molecular mechanisms underlying these parameters should identify markers that could be used as targets to modulate cell behaviour. This raises the fundamental new possibility that one could predict the ability of tissues to contract and scar and thus be able to anticipate and modulate tissue damage, which would have broad clinical implications.
